# Effect of Ultrasonication on Physical Properties of Mineral Trioxide Aggregate

**DOI:** 10.1155/2014/191984

**Published:** 2014-04-01

**Authors:** Peter Parashos, Amanda Phoon, Chankhrit Sathorn

**Affiliations:** Melbourne Dental School, University of Melbourne, 720 Swanston Street, Melbourne, VIC 3010, Australia

## Abstract

*Aim*. To evaluate the effect on physical properties of Mineral Trioxide Aggregate (MTA) of using direct hand compaction during placement and when using hand compaction with indirect ultrasonic activation with different application times. *Methods*. One hundred acrylic canals were obturated in 3 increments with MTA in sample sizes of 10. One group was obturated by hand with an endodontic plugger and the remainder obturated with indirect ultrasonic application, with times ranging from 2 seconds to 18 seconds per increment. Microhardness values, dye penetration depths, and radiographs of the samples were evaluated. *Results*. As ultrasonic application time per increment increased, microhardness values fell significantly (*P* < 0.001) while dye penetration values increased (*P* < 0.001). Microhardness of MTA ultrasonicated for 2 seconds was significantly higher than hand compaction (*P* = 0.03). Most radiographic voids were visible in the hand-compacted group (*P* < 0.001), which also had higher dye penetration depths than the 2-second ultrasonicated samples. Ultrasonication of MTA for 10–18 seconds resulted in significantly more voids than 2–8 seconds of ultrasonication (*P* = 0.02). *Conclusion*. The use of ultrasonics with MTA improved the compaction and flow of MTA, but excessive ultrasonication adversely affected MTA properties. A time of 2 seconds of ultrasonication per increment presented the best compromise between microhardness values, dye penetration depths, and lack of radiographic voids.

## 1. Introduction

MTA has grown in popularity as a dental material because of its largely favourable properties, including tissue biocompatibility, superior sealing ability, and its ability to promote dental pulp and periradicular tissue healing [1]. However, there have been concerns amongst clinicians about its difficult handling characteristics. It has been reported that MTA can have different physical and mechanical properties and lose consistency in the presence of excess liquid, which can occur even at the proportion recommended by manufacturers [2] and the resultant mix can lack adequate viscosity [3]. The long setting time of MTA results in an “initial looseness which can make handling rather difficult” [4]. An effective intracanal placement technique of a material such as MTA is imperative, and improving its delivery technique is the key to enhancing MTA's favourable properties [5]. Because there have been few studies examining the variations of placement techniques of MTA, there is little applied consistency amongst its users. There seems to be limited, and contradictory, information on methods to best handle MTA.

Ultrasonics in dentistry has a wide range of applications and has arguably improved treatment outcomes and predictability [6]. Ultrasonic vibration applied to an endodontic condenser aims to improve the flow, settling, and compaction of MTA and is perceived to be a useful adjunct [7]. However, ultrasonic activation of MTA has been found to result in more voids and poorer adaptation [8]. Furthermore, ultrasonically overcompacted MTA may show poorer physical characteristics because excessive ultrasonication may incorporate air into the MTA and produce a fill less dense and less uniform than that produced by hand compaction [9]. Similarly, El-Ma'aita et al. [10] found denser MTA root fillings with manual compaction than with ultrasonic activation. However, regardless of mixing techniques, ultrasonically compacted MTA showed increased compressive strength compared with hand-mixed samples [11]. Ultrasonically compacted MTA produced much higher surface microhardness values than manually placed samples [12]. Another study has found significant effect on the push-out bond strength of MTA regardless of mixing methods or ultrasonic application [13].

These different and contradictory findings may be the result of different times used to ultrasonically compact MTA. Therefore, the aim of this study was to evaluate the effect of different ultrasonic application times on the density, compressive strength, and radiopacity of MTA.

## 2. Materials and Methods

Twenty uniform acrylic blocks of five canals each were prepared, creating 100 samples. Each simulated canal measured 1 mm in diameter by 6 mm in length. The canals in two of these blocks (*n* = 10) were obturated with MTA without ultrasonic activation. ProRoot MTA (Dentsply Maillefer, Ballaigues, Switzerland) was mixed according to manufacturer's instructions with the supplied sterile water in a 3 : 1 powder/liquid ratio [14] and hand compacted into the acrylic canals using a similarly sized stainless steel endodontic plugger (American Eagle, Missoula, MT, USA). Each canal was filled in three increments against a glass slab. Both blocks were then placed into a petri dish under cotton pellets soaked with room temperature distilled water and placed in an incubator at 37°C for 24 hours at 95% humidity.

All of the other samples (*n* = 90) were obturated with indirect ultrasonic activation against the endodontic plugger. Samples 11–20 were compacted with 2 seconds of ultrasonic activation, samples 21–30 for 4 seconds, samples 31–40 for 6 seconds, and so forth until samples 91–100, which were ultrasonically activated for 18 seconds. These samples were also filled in three increments, with an ultrasonic tip held lightly against the plugger at each increment for the desired time period. The Cavi 3D tip of the VDW Ultrasonic unit (Aceton, North America, NJ, USA) was used at the middle setting. The MTA fillings were flush with the surface of the acrylic block, and all blocks were placed into petri dishes under cotton pellets soaked with room temperature distilled water. All blocks were then left in an incubator at 37°C for 24 hours at 95% humidity.

## 3. Radiographs

Radiographs were taken for each acrylic block at a set distance of 50 centimeters to the cone of the X ray unit. The Sirona Heliodent DS X ray unit (Sirona Dental Systems, Bensheim, Germany) was set at 70 kV, a current of 7 mA, and an exposure time of 0.25 seconds. These radiographs were then examined for voids, which presented as clearly demarcated radiolucent areas within the specimens. The number of voids, when present, was counted in each specimen.

## 4. Microhardness

The Vickers microhardness was evaluated for the 100 samples. After 24 hours, all the blocks were lightly lapped with 400-grit fine sandpaper to produce a smooth surface, and one cement surface per canal was loaded with one Newton for six seconds, with a slope of 0.99. This resulted in a stamp indent on the MTA surface, with an impression of two orthogonal diagonals. An image of this was brought into focus using the computer and captured immediately after discharge of the diamond indenter. The microhardness of each sample was evaluated using standard calculations [15].

## 5. Dye Penetration

The samples were left in a sealed container for an additional 24 hours after the microhardness testing and immersed in a 0.2% Rhodamine Blue solution (Ryond Chemical Company, Tianjin, China) for 72 hours in the incubator and then visually assessed for dye penetration. Each of the 100 specimens was examined under a light microscope with 4x magnification and a Michigan “O” cc periodontal probe (Hu-Friedy, Rotterdam, Netherlands) was used to measure the depth of dye penetration. The periodontal probe was held against each specimen and measured to the nearest 0.5 mm.

Statistical analysis included Pearson Correlations, two-sample *t*-test, Regression analysis, and Fisher's exact test with significance set at *P* < 0.05.

## 6. Results

**Figure 1:**
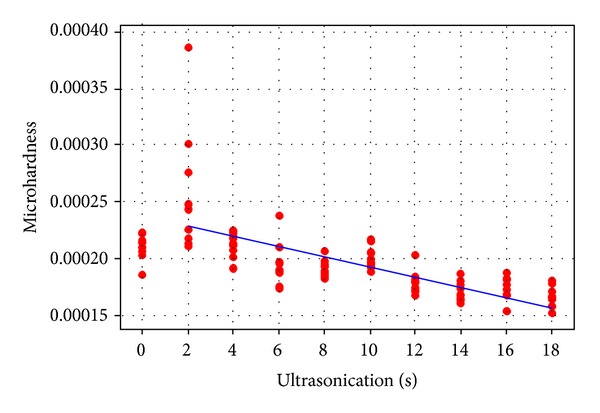
Microhardness values and ultrasonication time.

**Figure 2:**
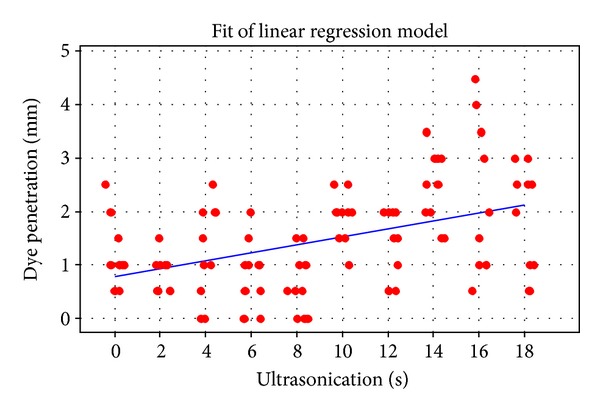
Dye penetration and ultrasonication time.

**Figure 3:**
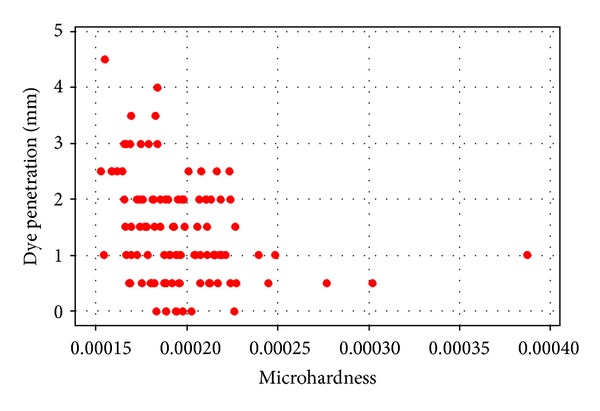
Dye penetration and microhardness.

Evaluation of the microhardness (Figure 1) showed a correlation between increased ultrasonication times and the microhardness of the MTA samples. An approximately linear correlation was demonstrated, with increasing times of ultrasonication resulting in a lower microhardness value (*r* = −0.707, where *r* is the correlation coefficient; *P* < 0.001). A two-sample *t*-test indicated a significant difference (*P* = 0.03) between hand compaction and two-second ultrasonication. As ultrasonication time increased, the dye penetration of the MTA samples (Figure 2) also increased in an approximately linear manner (*r* = 0.446, *P* < 0.001). As dye penetration increased (Figure 3), a corresponding fall in microhardness values was also seen (*P* = 0.001).

When viewing the radiographs taken of the acrylic blocks, a significantly higher proportion (*P* < 0.001) of visible voids were noted in the hand-compacted group (90%) than in the ultrasonicated groups overall (22%). Grouping and dichotomising of the ultrasonication groups only indicated a significantly lower proportion (*P* = 0.02) of groups with voids when ultrasonicated for 2–8 seconds (10%) than 10–18 (54%) seconds.

## 7. Discussion

Hydration of the MTA powder results in a colloidal gel that solidifies to a hard structure in less than three hours, which then continues to set [16]. The basic framework of the hydrated mass is formed by the interlocking of cubic and needle-like crystals in which the needle-like crystals form in sharply delineated thick bundles that fill the intergrain space between the cubic crystals [17]. Its resultant characteristics depend upon liquid additives and quantity [17], as well as entrapped air [18], which may be recognized radiographically. In the present study it was observed that the hand-compacted samples showed more radiographic voids, which was contrary to the findings of Aminoshariae et al. [8]. However, their method differed with the current study because they used 10 mm lengths of MTA and up to 30 seconds of ultrasonication, although the latter time was not actually specified. Nevertheless, those authors speculated that the reason for their results may have been the length and method of MTA placement [8]. Possibly, the shorter length of MTA and shorter ultrasonication time of the current study were responsible for the better outcome.

In another comparison of hand and ultrasonic compaction [9] ultrasonication produced a denser MTA fill. This study used an ultrasonication time of only one second after their pilot study indicated that up to five-second ultrasonication resulted in radiographically detectable voids. Another recent study [10] used micro-CT analysis and found fewer voids with hand compaction but decreasing number of voids with longer ultrasonication times of five or ten seconds. The present study was unique in assessing a broad range of ultrasonication times, which showed deterioration of the physical properties tested. This may support the phenomenon described by El-Ma'aita et al. [10] where prolonged activation times may rearrange the MTA particles leading to changes in the numbers of voids, density, and microhardness. Conceivably, the frequency and method of ultrasonication would also exert an effect on the MTA particle arrangement.

The Vickers microhardness test used in this study aimed to quantify the resistance of MTA to plastic deformation. While the compressive strength helps to indicate the setting and hydration reaction progress [19, 20], microhardness in itself is not a fundamental material property. In this case, it served to provide a means of assessing the MTA properties between the different samples, and the correlation found was that with increased times of ultrasonication, sample microhardness values fell. Similarly, longer activation time has been found to produce more voids and a lower weight of MTA leading to less dense compactions [9]. This may correlate with the lower microhardness values found in the present study.

The method used for the dye penetration study was adapted from that of Bortoluzzi et al. [21] and aimed to evaluate the resistance of MTA to ingress of fluid. It is recognised that dye studies measuring leakage along root fillings are unreliable and lead to questionable results in extracted teeth [22]. However, in the current study, the amount of dye penetration was used to measure differences of fluid ingress against a smooth plastic wall after different periods of ultrasonication with the aim of assessing potential disturbance of the physical properties of the MTA rather than leakage per se. The hand-compacted samples showed deeper dye penetration. In a similar manner to the microhardness values, with increased ultrasonication of the samples, dye penetration depths rose. This correlates with the number of voids in the MTA, which would influence dye penetration rates. However, it should be noted that the standard deviations in each group were quite wide which may have reflected the measurement method using the periodontal probe and hence some imprecision.

Overall, the results indicated that an ultrasonication time of 2 seconds was better than hand compaction. The higher depth of dye penetration and the higher incidence of radiographic voids amongst the hand-compacted samples indicate that the use of indirect ultrasonic activation is beneficial. It is difficult to directly compare the results of the present study with the available literature because they differ in too many aspects, including type of MTA, the assessment method, length of MTA fill, type of canal used, canal dimensions, ultrasonication time, and ultrasonic unit used. Parameters measured also differ and include bacterial leakage [5], push-out strengths [13], solubility [3, 23], fracture resistance, and compressive strength [7, 11].

## 8. Conclusion

The use of ultrasonics with MTA was useful in improving flow and compaction of MTA, but excessive ultrasonication can adversely impact MTA properties. A suggested time of 2 seconds of ultrasonication per increment presented the best compromise between microhardness values, dye penetration depths, and lack of radiographic voids.
